# Supplementary figures and images for: Impact of the clinically approved BTK inhibitors on the conformation of full-length BTK and analysis of the development of BTK resistance mutations in chronic lymphocytic leukemia
Source: eLife. 2024 Dec 27;13:RP95488. doi: 10.7554/eLife.95488 (PMC11677227; doi:10.7554/eLife.95488)

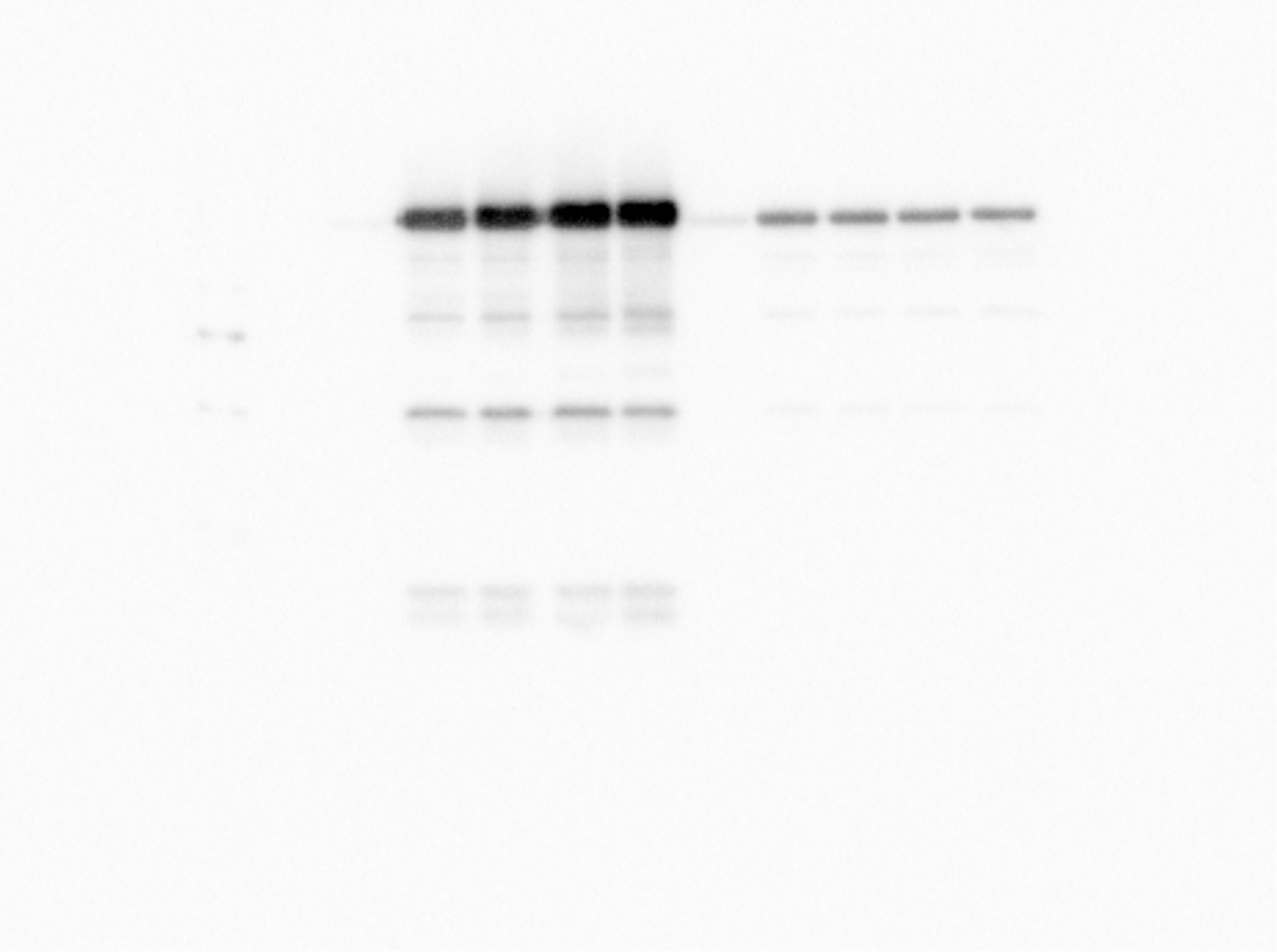

Supplement: Figure 6—source data 1. [file elife-95488-fig6-data1.zip › Figure6a_Anti_BTK_pY551blot.tif]

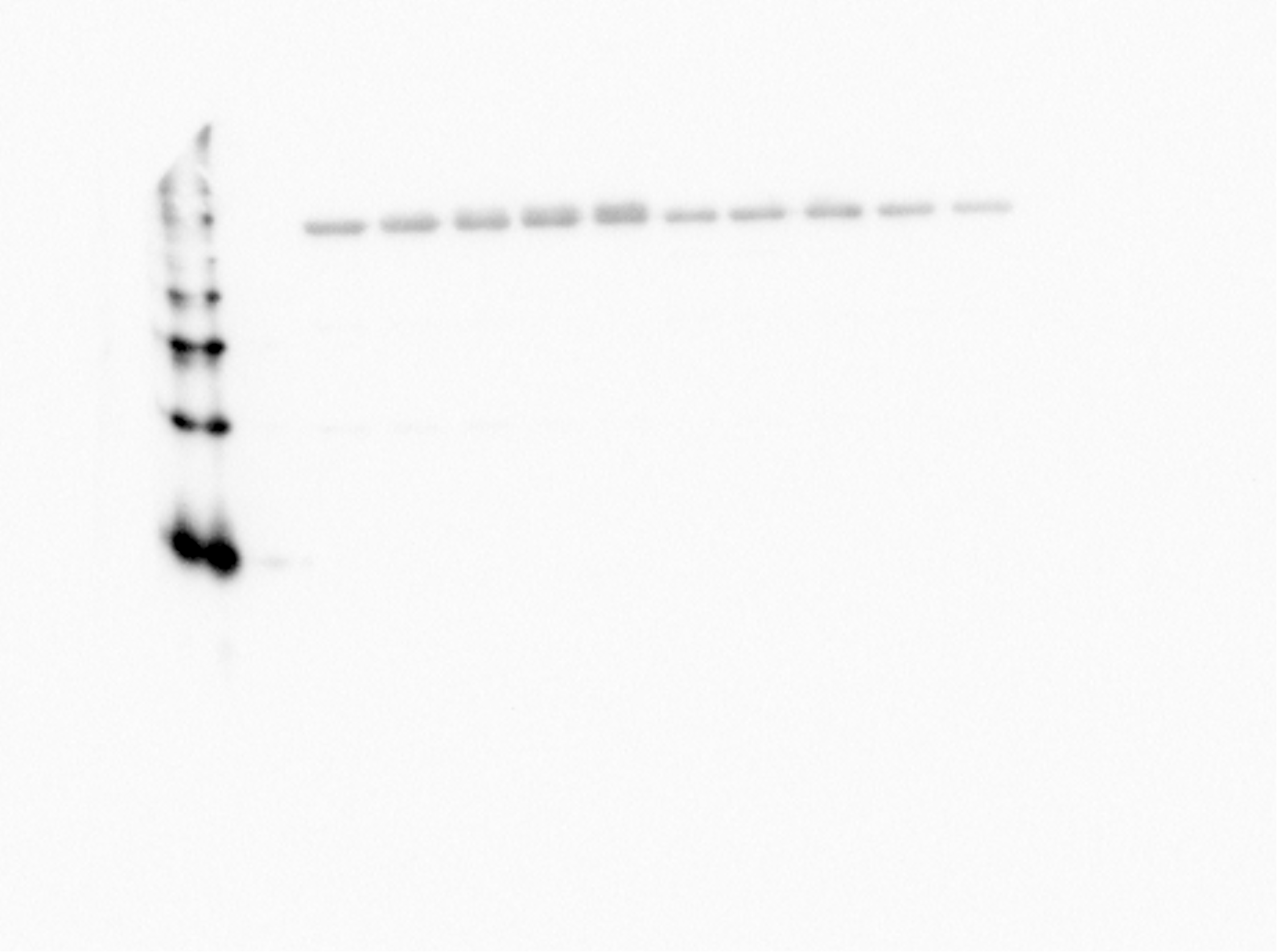

Supplement: Figure 6—source data 1. [file elife-95488-fig6-data1.zip › Figure6a_Anti_Hisblot.tif]

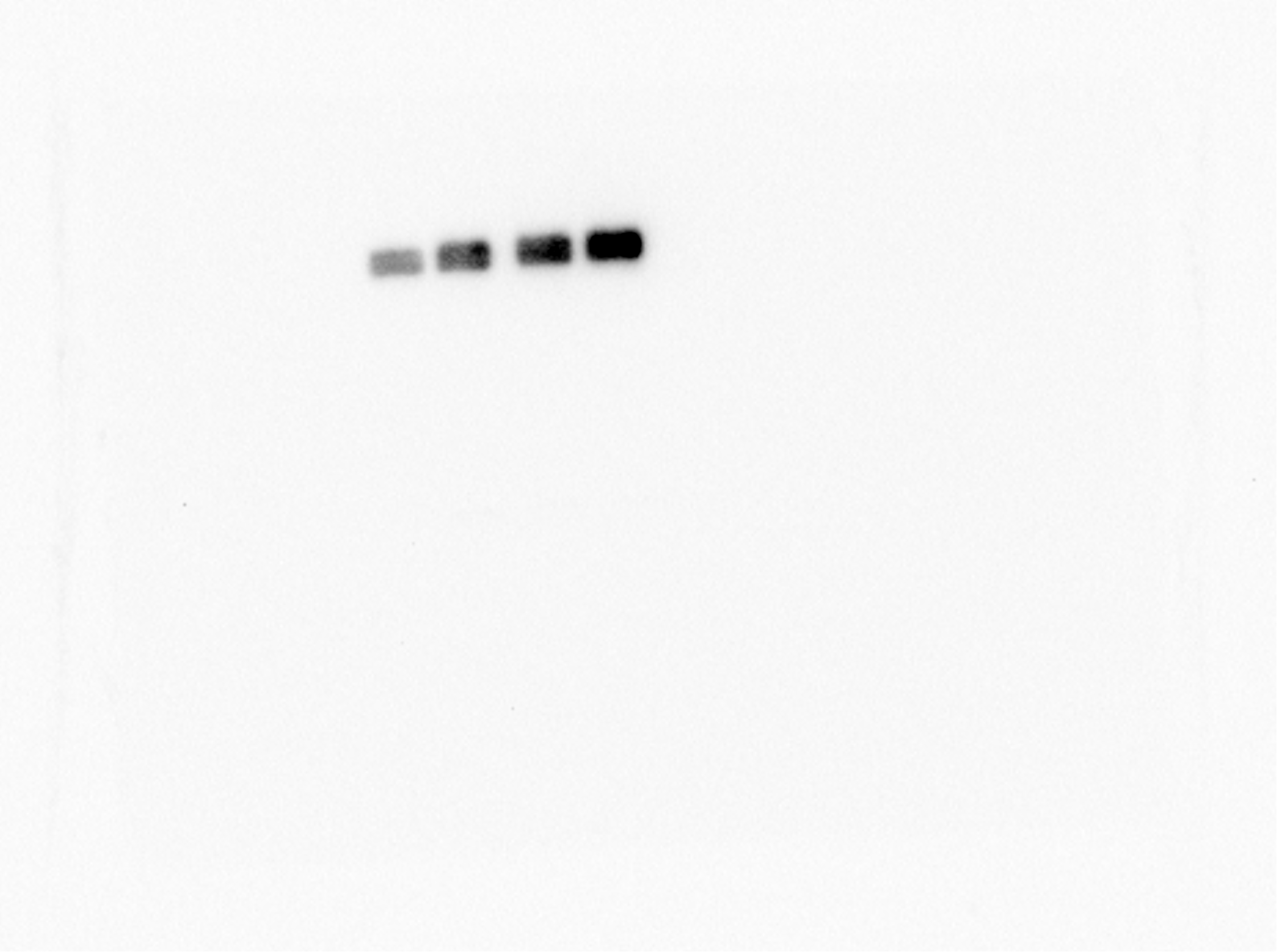

Supplement: Figure 6—source data 3. [file elife-95488-fig6-data3.zip › Figure6c_Anti_BTK_pY551blot.tif]

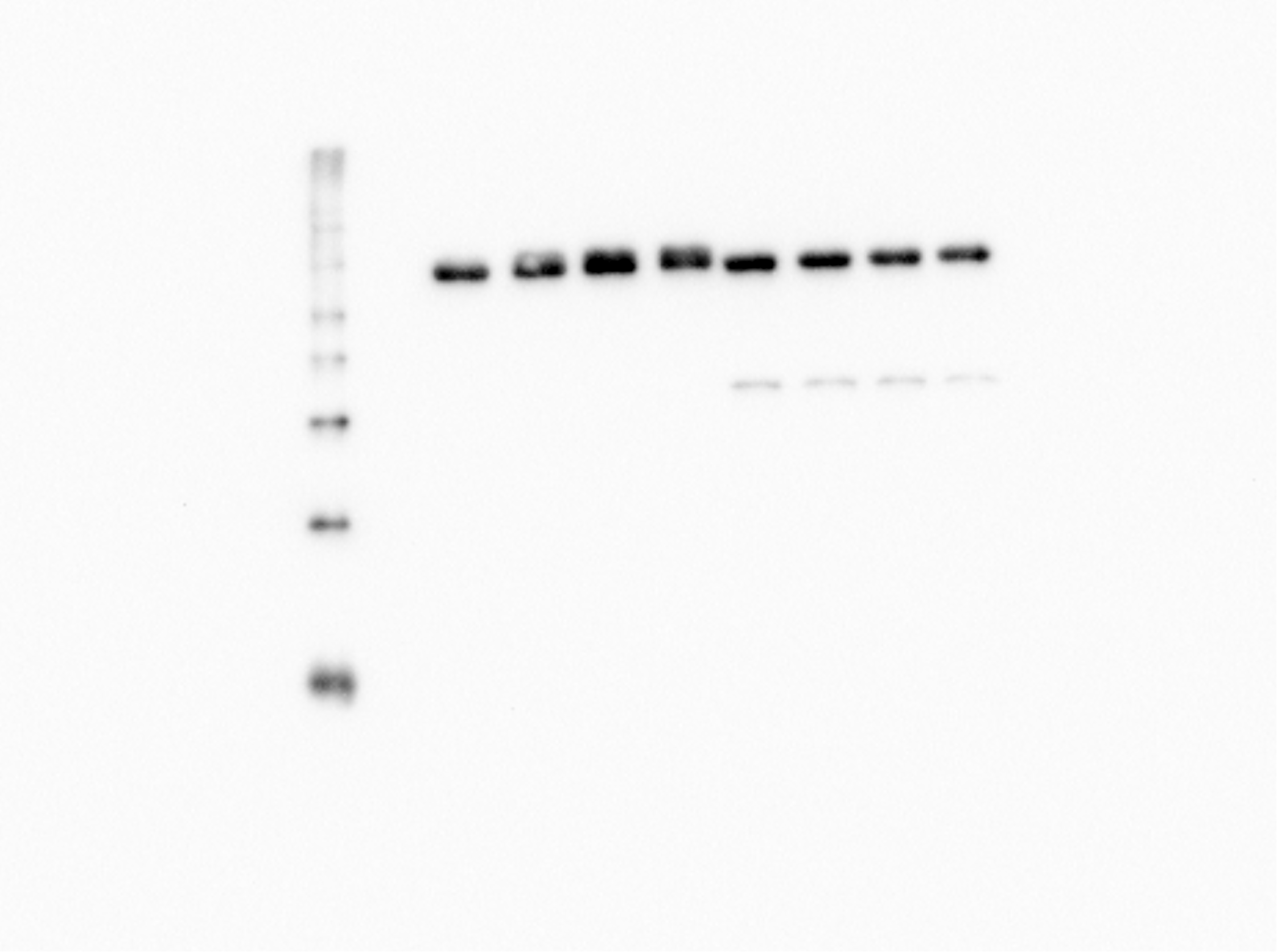

Supplement: Figure 6—source data 3. [file elife-95488-fig6-data3.zip › Figure6c_Anti_Hisblot.tif]

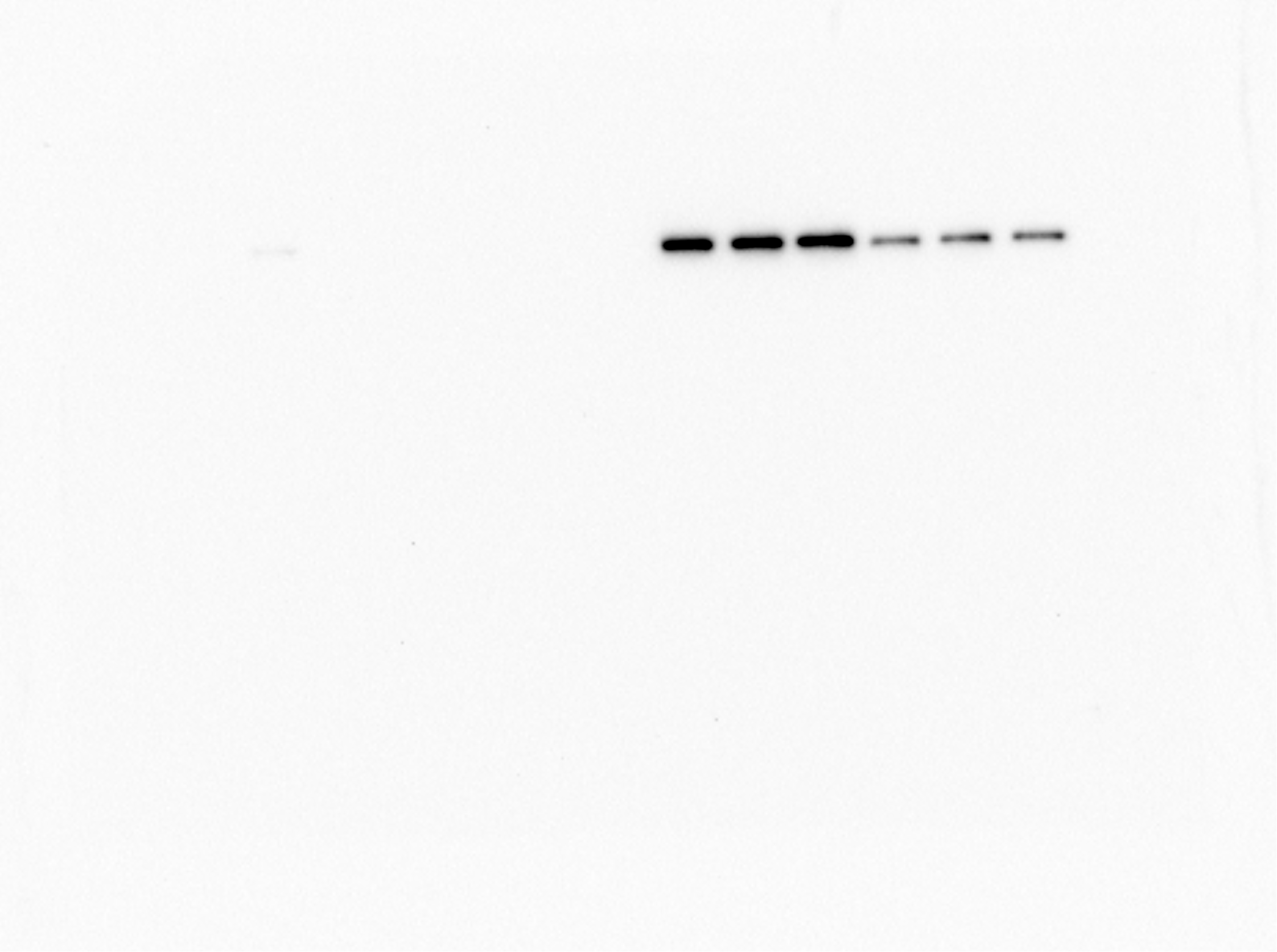

Supplement: Figure 7—source data 1. [file elife-95488-fig7-data1.zip › Figure7a_AntiBTK_pY551.tif]

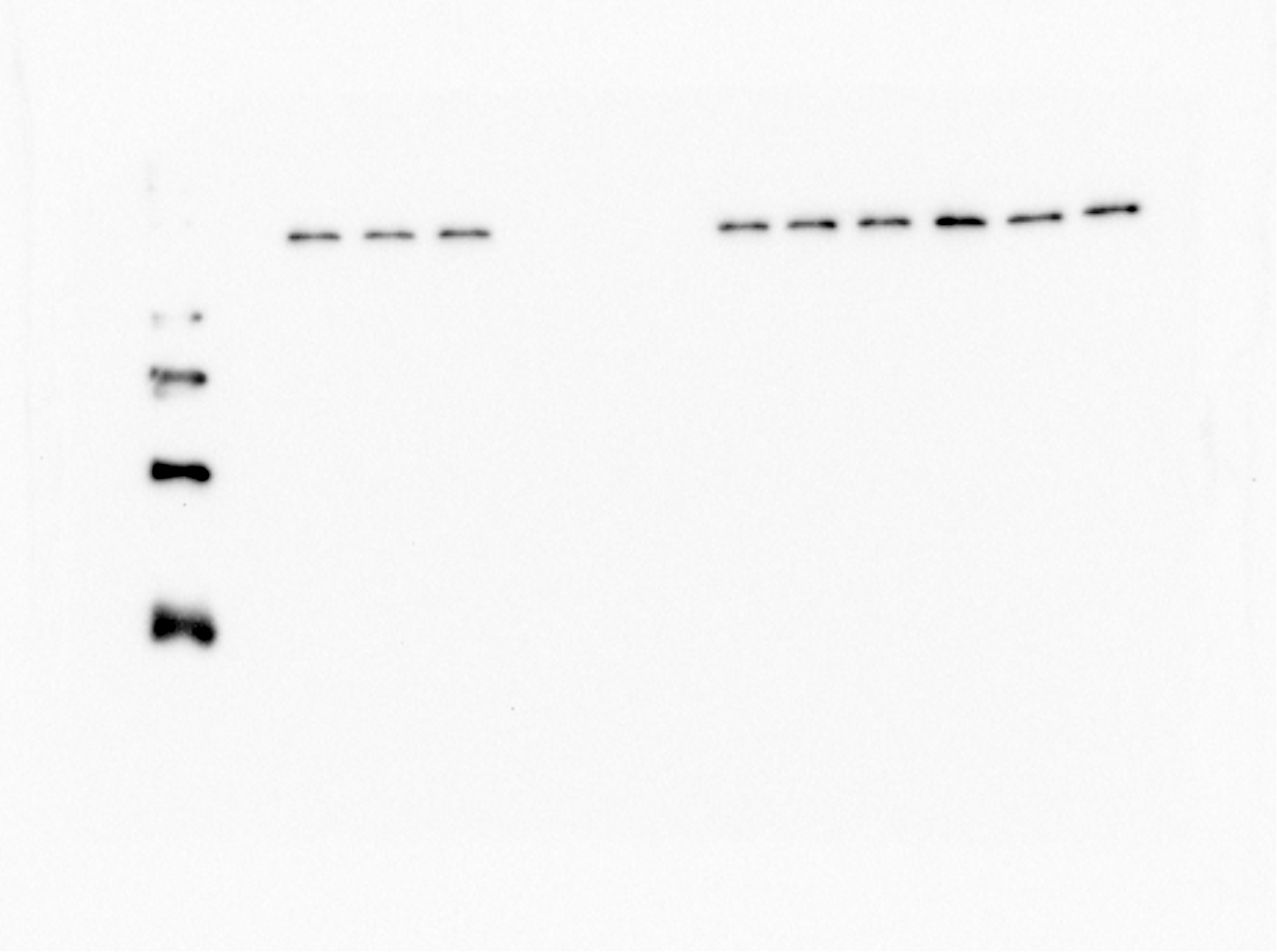

Supplement: Figure 7—source data 1. [file elife-95488-fig7-data1.zip › Figure7a_AntiHisblot.tif]

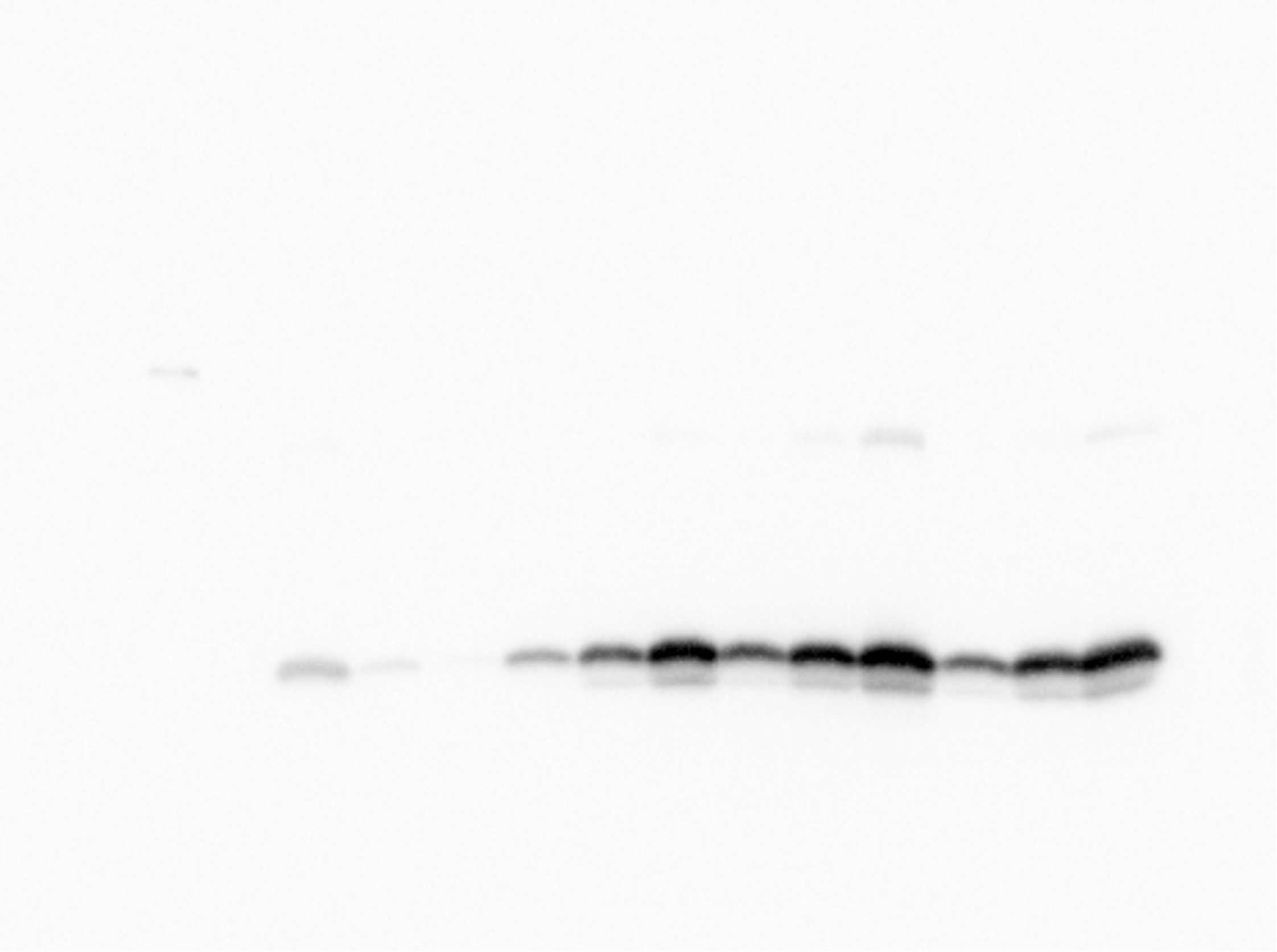

Supplement: Figure 7—source data 1. [file elife-95488-fig7-data1.zip › Figure7a_AntiPLCg_pY783blot.tif]

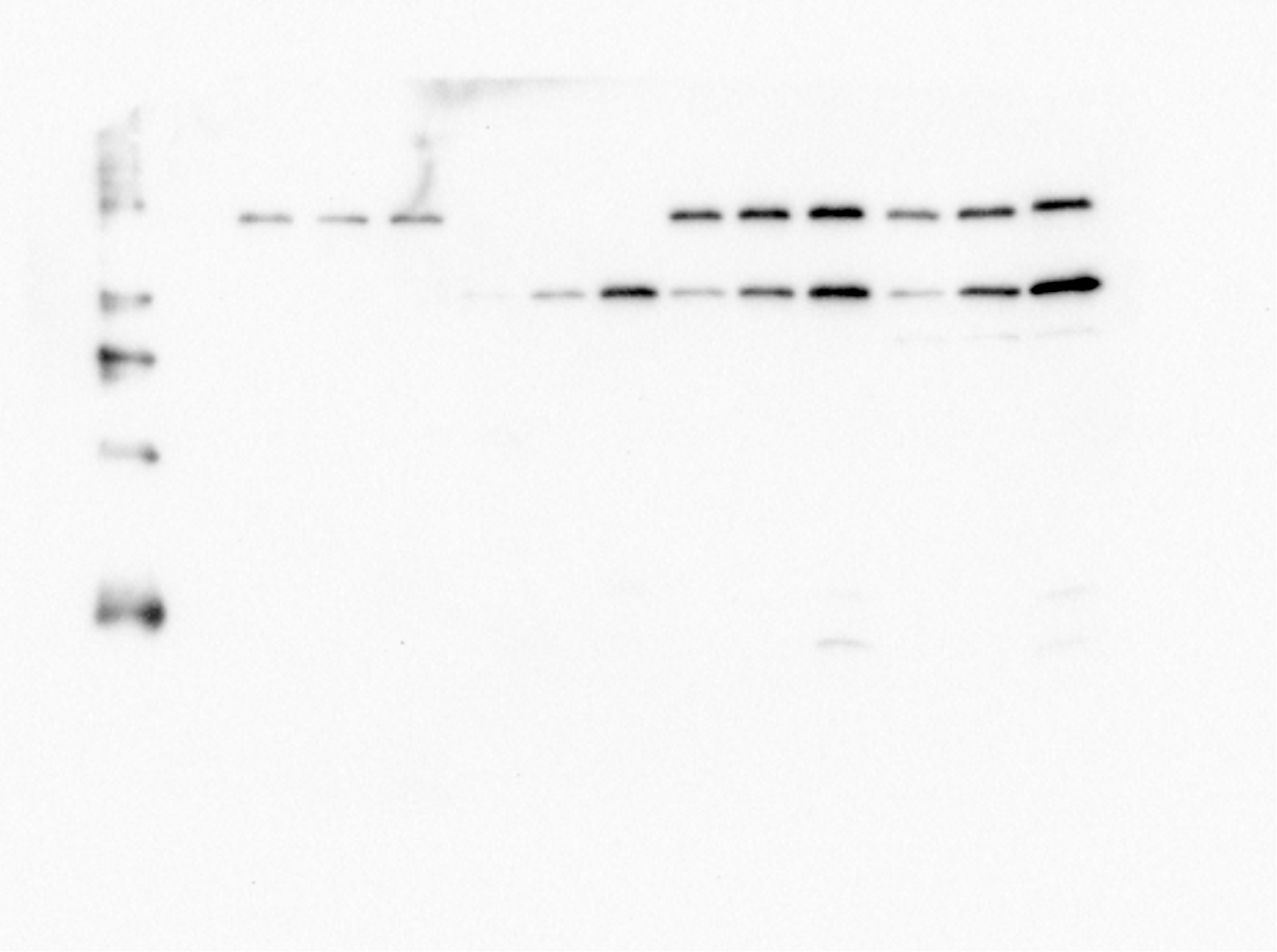

Supplement: Figure 7—source data 1. [file elife-95488-fig7-data1.zip › Figure7a_Anti_pYblot.tif]

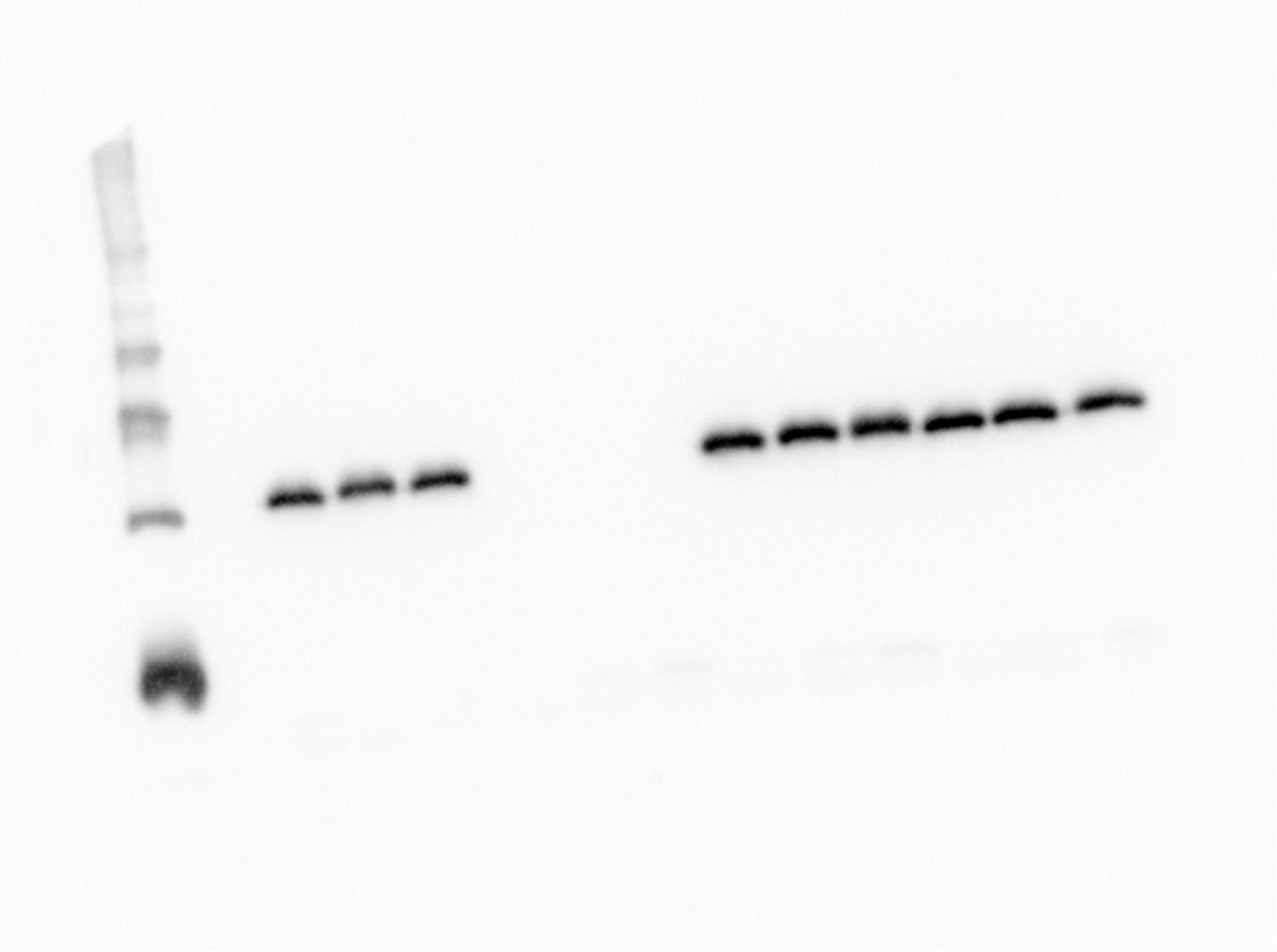

Supplement: Figure 7—source data 3. [file elife-95488-fig7-data3.zip › Figure7c_Anti_Hisblot.tif]

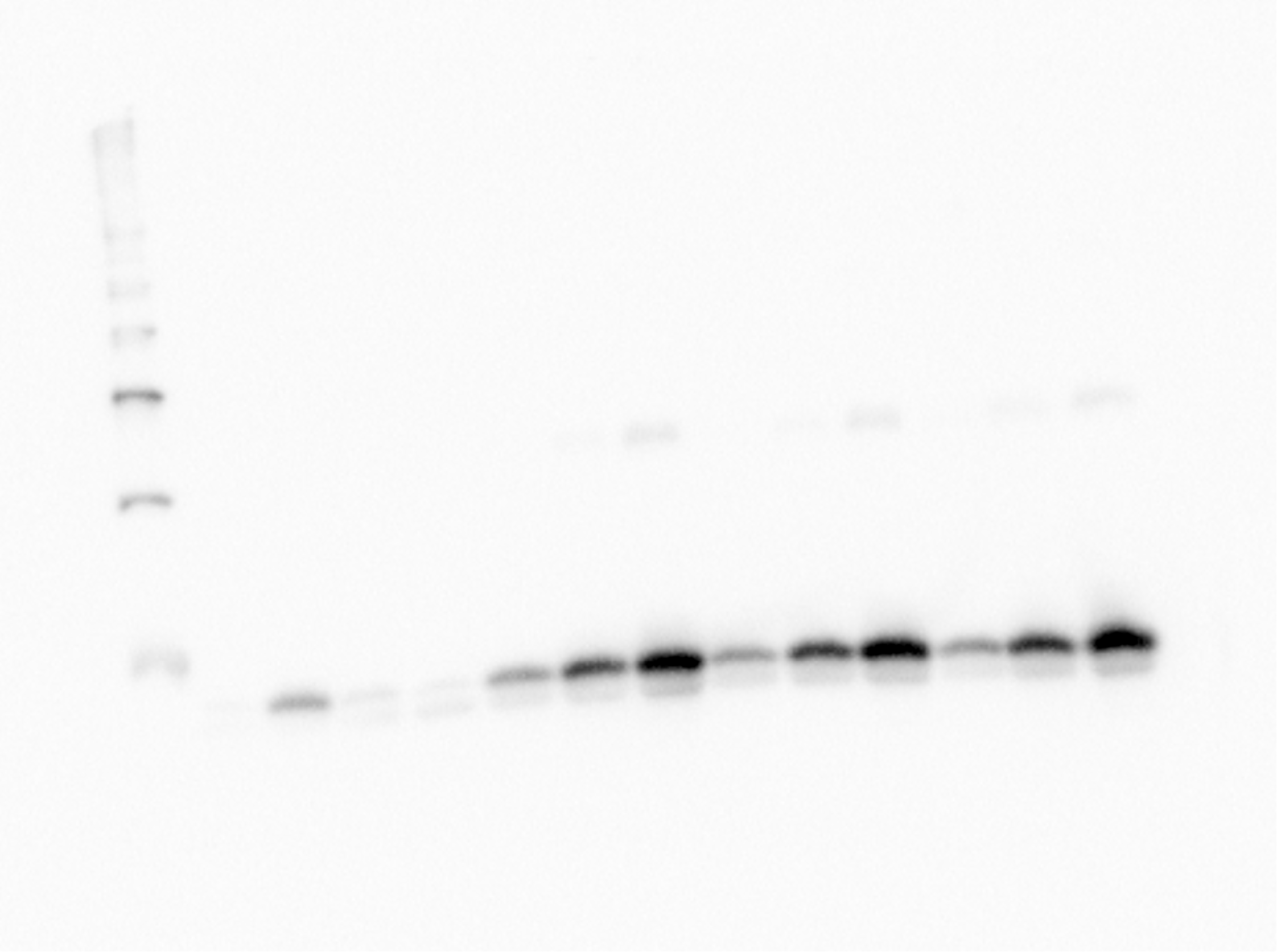

Supplement: Figure 7—source data 3. [file elife-95488-fig7-data3.zip › Figure7c_Anti_PLCg_pY783blot.tif]

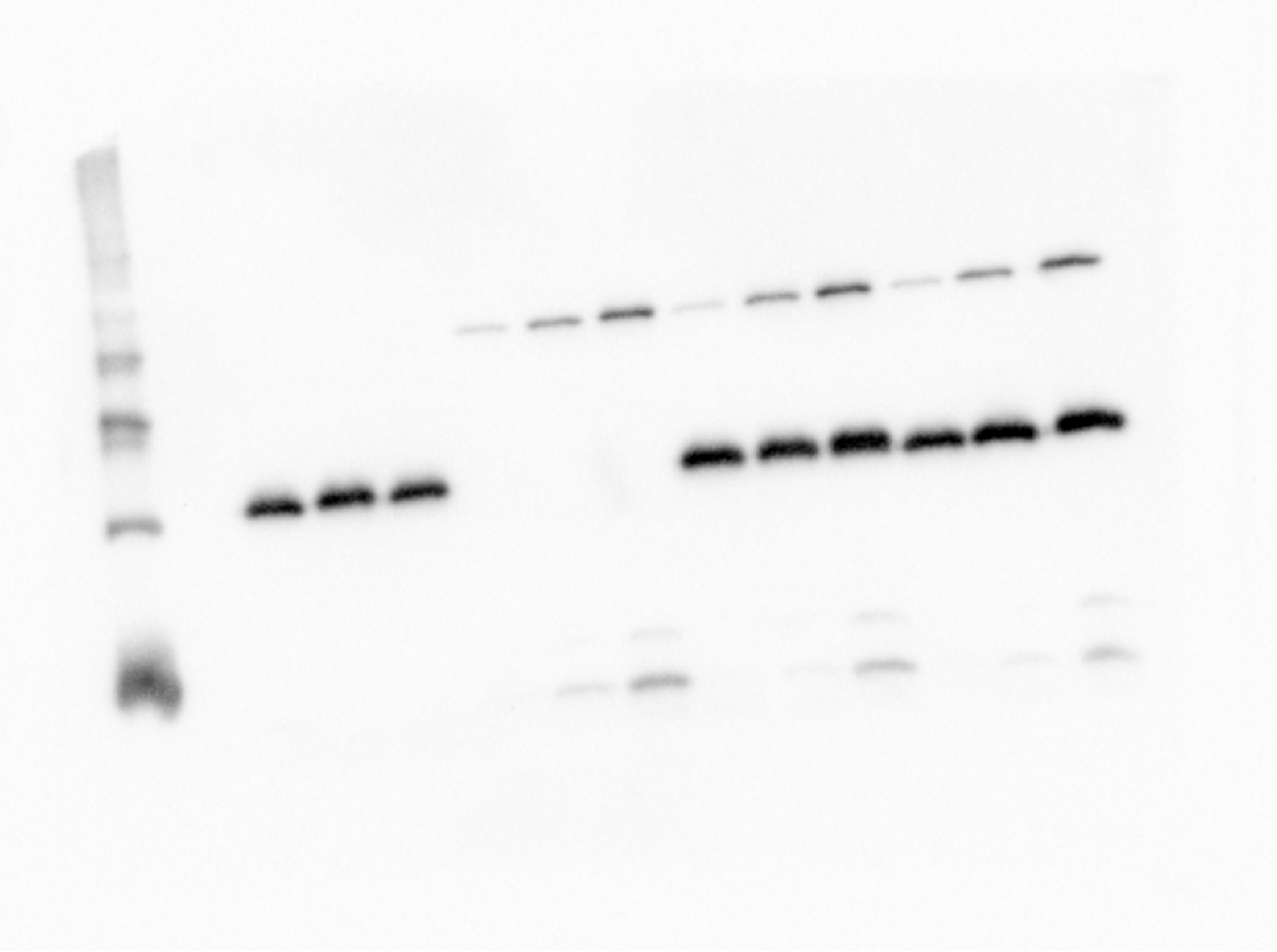

Supplement: Figure 7—source data 3. [file elife-95488-fig7-data3.zip › Figure7c_Anti_pYblot.tif]

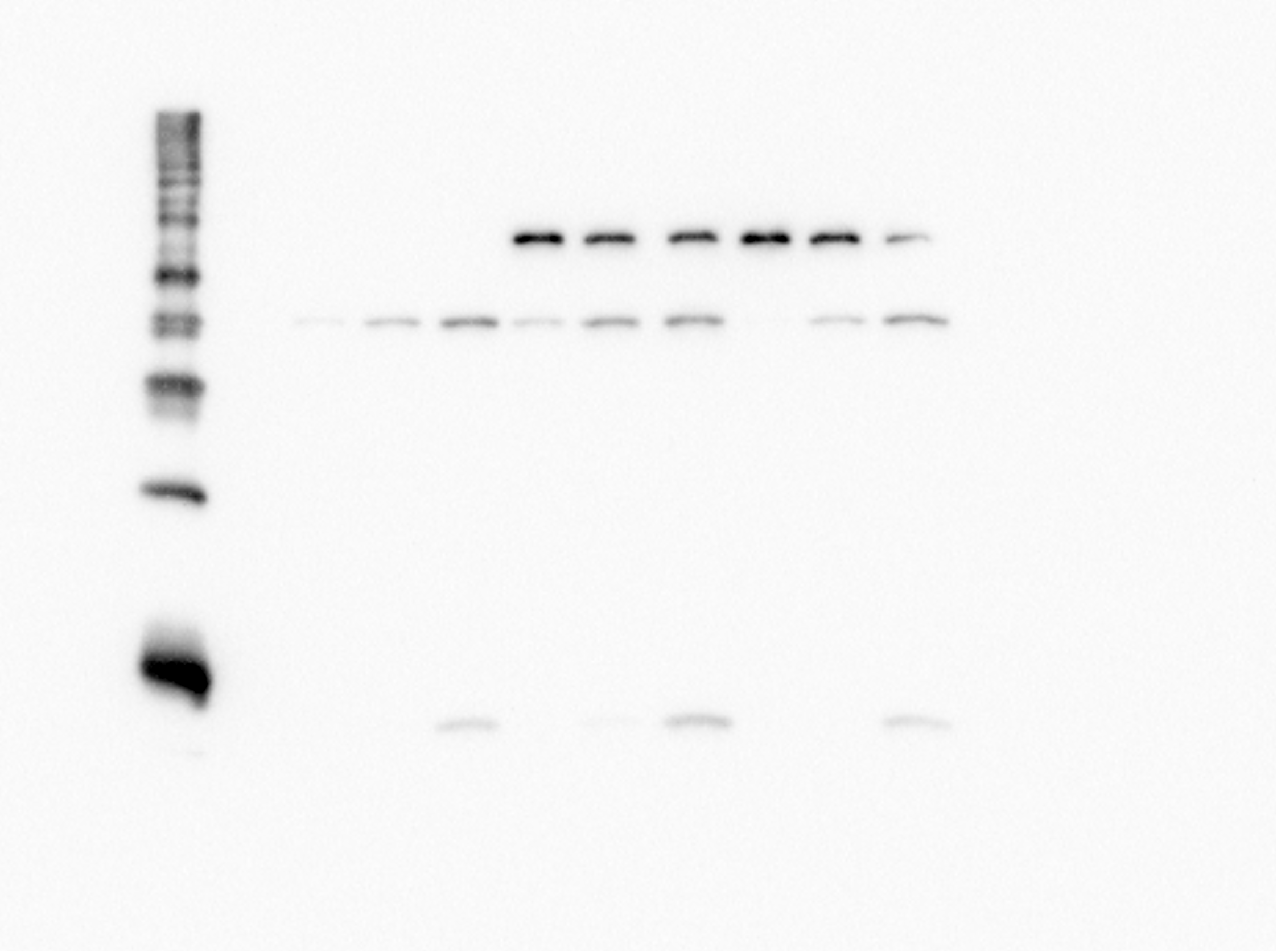

Supplement: Figure 7—source data 5. [file elife-95488-fig7-data5.zip › Figure7e_Anti_Hisblot.tif]

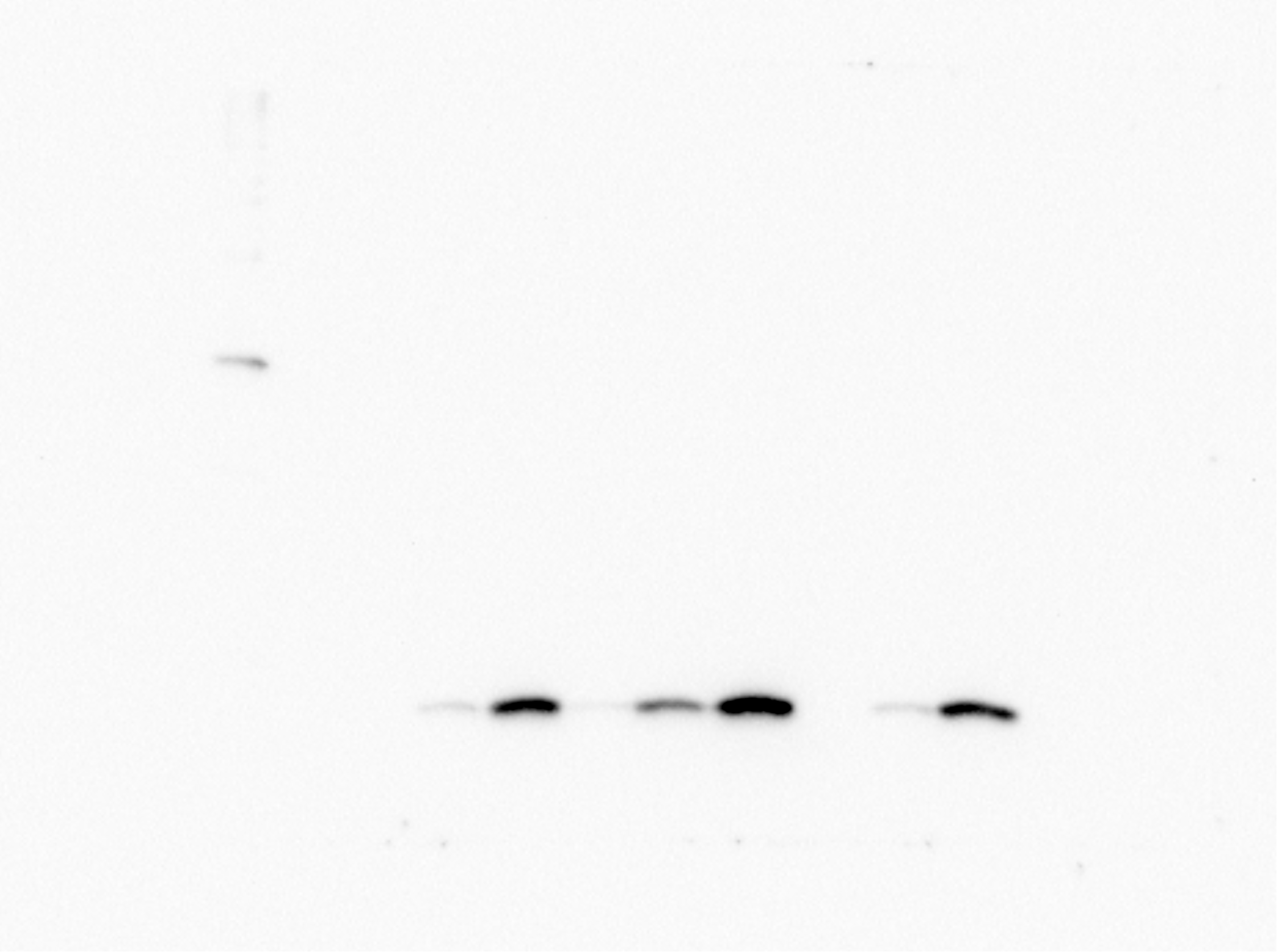

Supplement: Figure 7—source data 5. [file elife-95488-fig7-data5.zip › Figure7e_Anti_PLCg_pY783blot.tif]

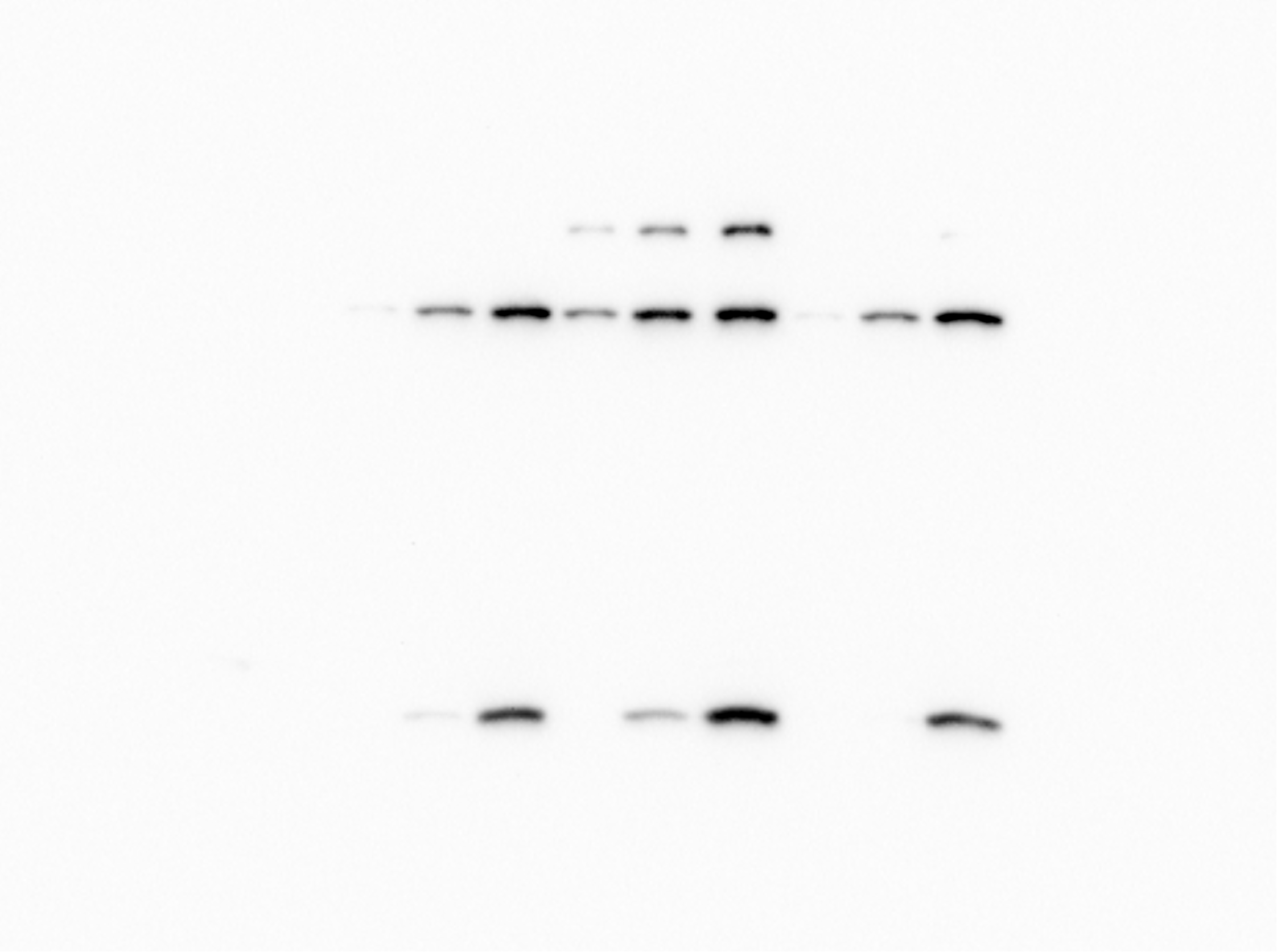

Supplement: Figure 7—source data 5. [file elife-95488-fig7-data5.zip › Figure7e_Anti_pYblot.tif]

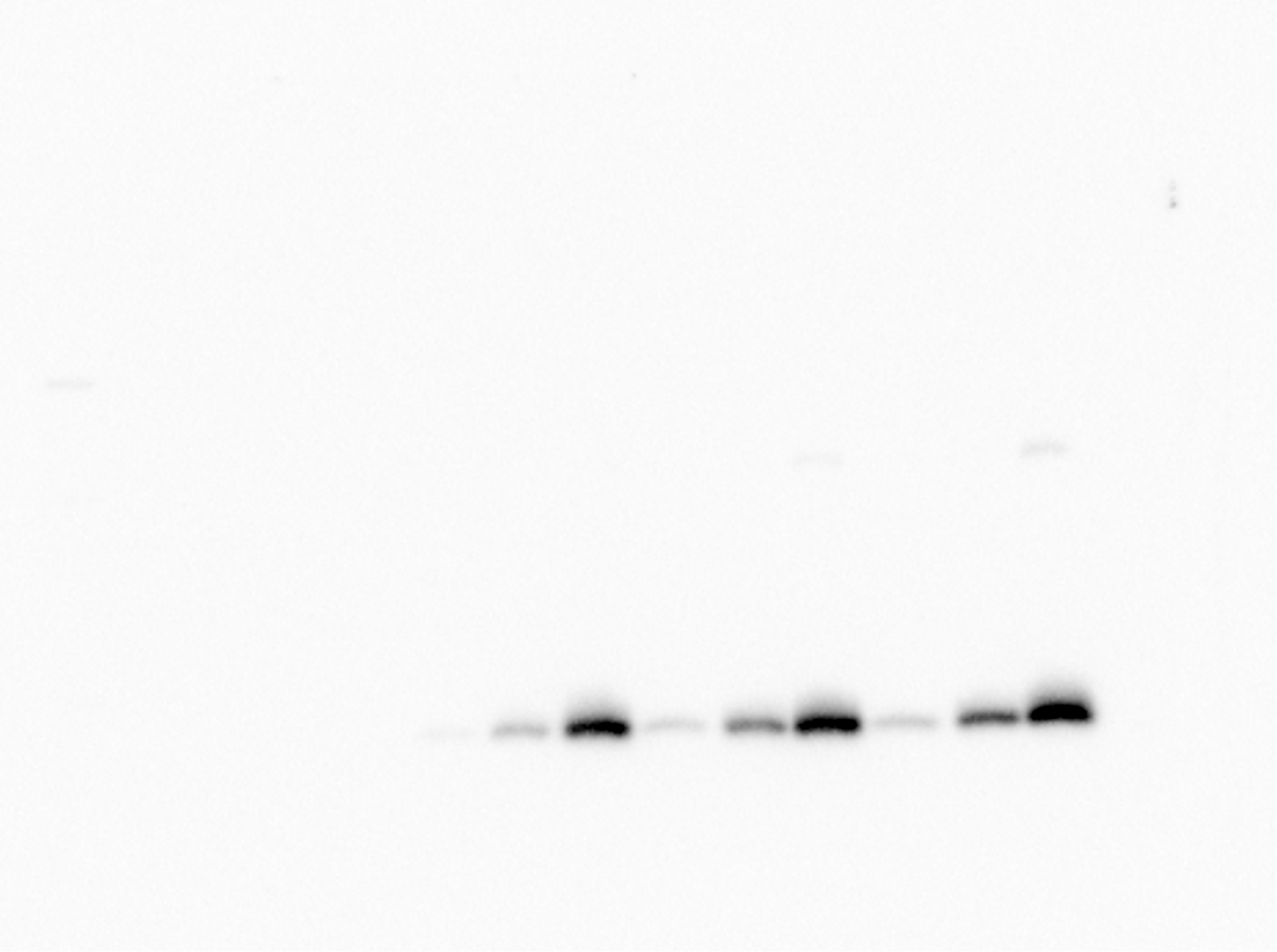

Supplement: Figure 7—figure supplement 1—source data 1. [file elife-95488-fig7-figsupp1-data1.zip › Fig7_Fig_supp1a_Anti_PLCg_pY783.tif]

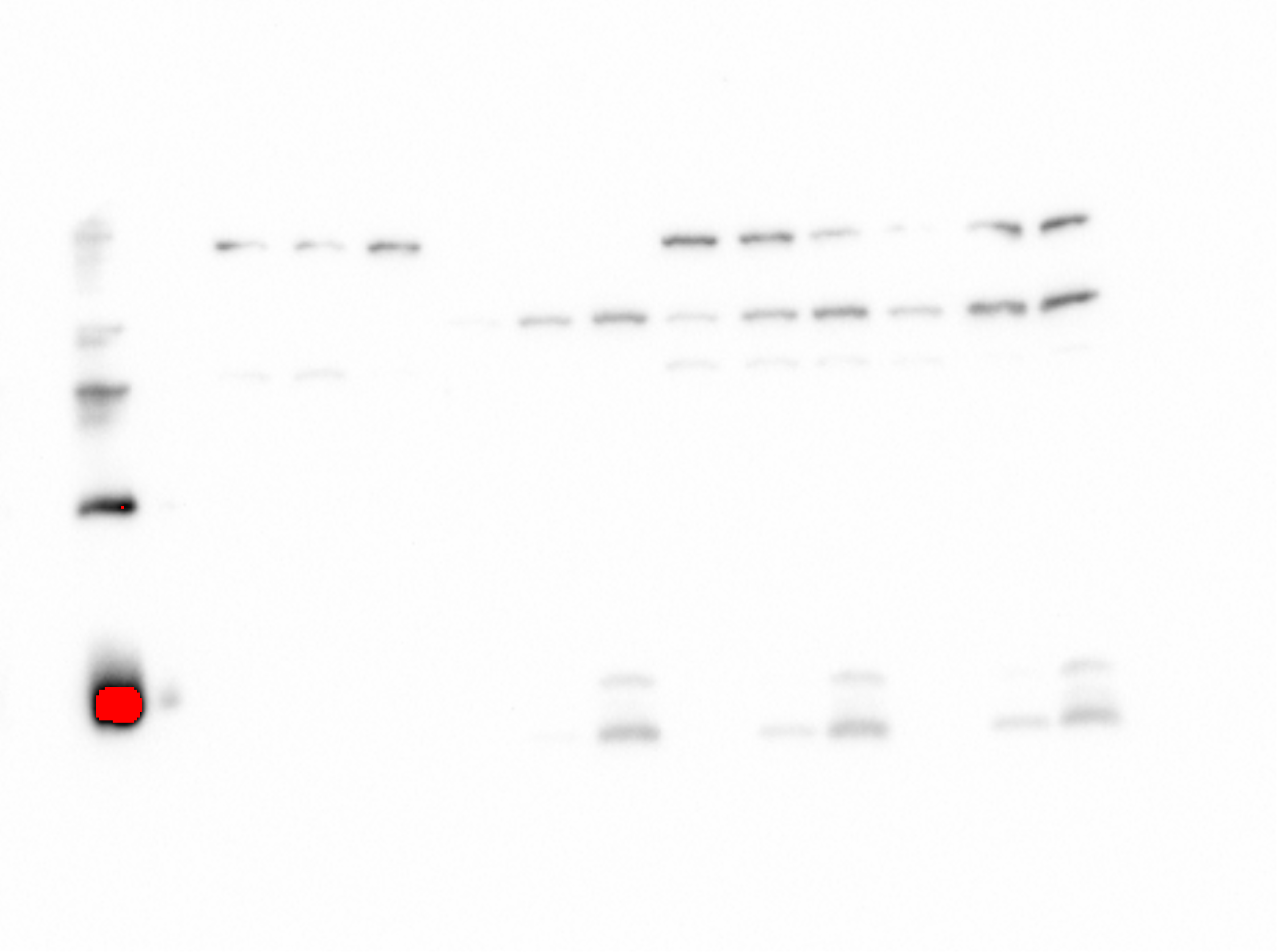

Supplement: Figure 7—figure supplement 1—source data 1. [file elife-95488-fig7-figsupp1-data1.zip › Figure7_figure_supplement1a_Anti_Hisblot.tif]

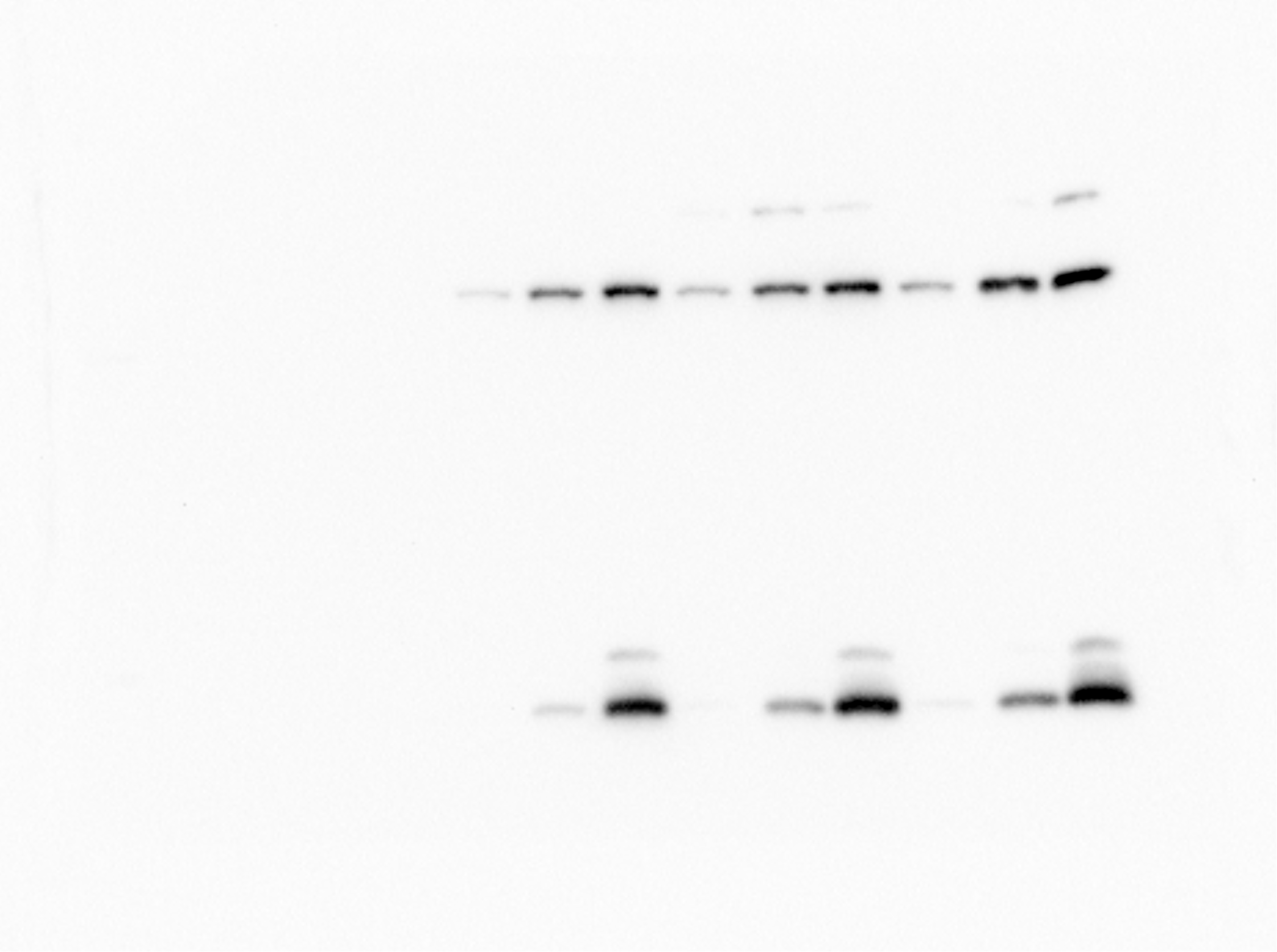

Supplement: Figure 7—figure supplement 1—source data 1. [file elife-95488-fig7-figsupp1-data1.zip › Figure7_figure_supplement1a_Anti_pYblot.tif]

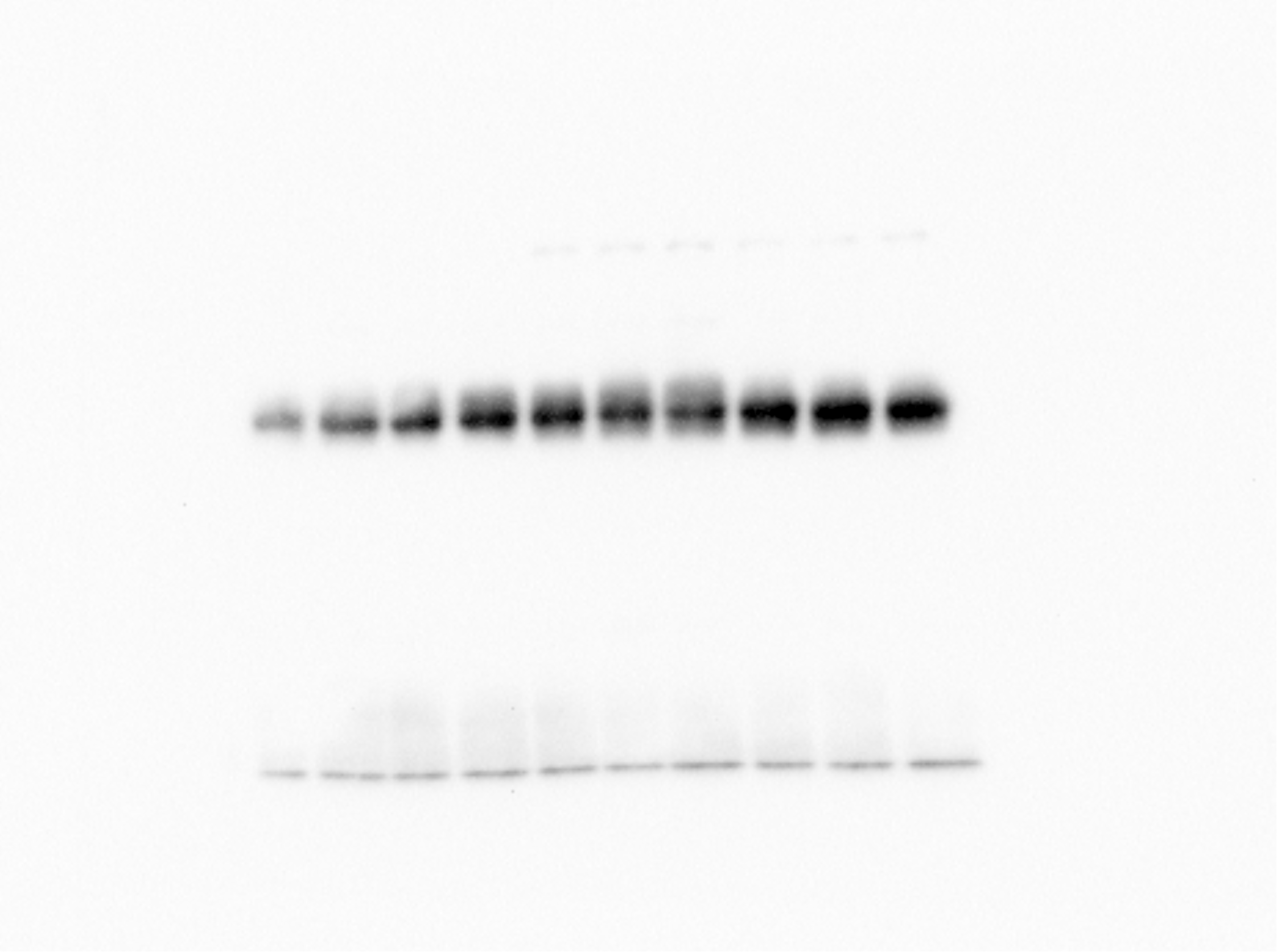

Supplement: Figure 7—figure supplement 1—source data 3. [file elife-95488-fig7-figsupp1-data3.zip › Figure7_figure_supplement1b_Anti_Hisblot.tif]

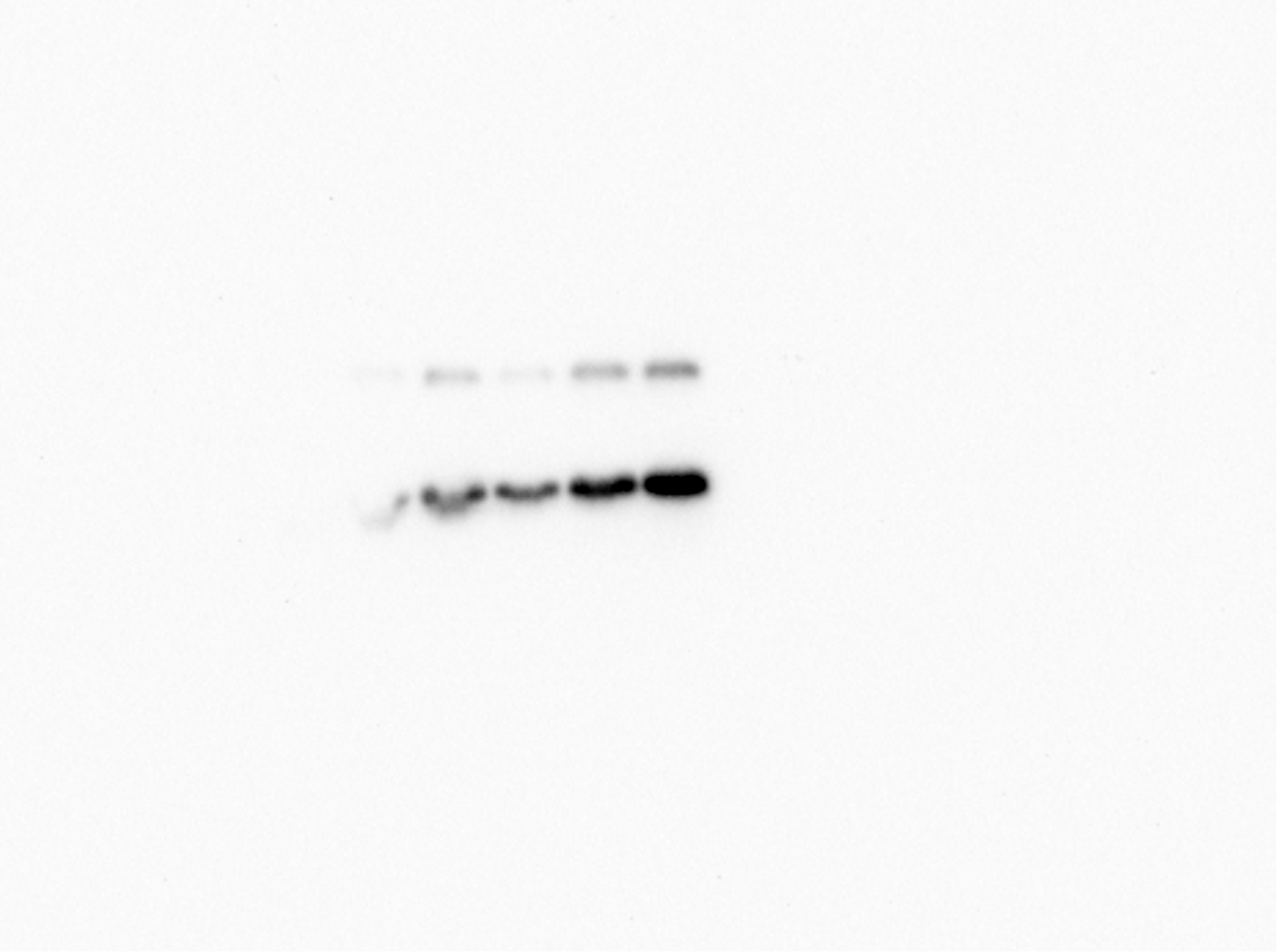

Supplement: Figure 7—figure supplement 1—source data 3. [file elife-95488-fig7-figsupp1-data3.zip › Figure7_figure_supplement1b_Anti_pYblot.tif]
